# Effectiveness of AMD3100 in treatment of leukemia and solid tumors: from original discovery to use in current clinical practice

**DOI:** 10.1186/s40164-016-0050-5

**Published:** 2016-07-16

**Authors:** Tao Liu, Xiaobo Li, Shuo You, Soumitra S. Bhuyan, Lei Dong

**Affiliations:** 1 Division of Hematology/Oncology, Department of Pediatrics, Aflac Cancer and Blood Disorders Center, Emory University School of Medicine, 1760 Haygood Drive NE, HSRB E363, Atlanta, GA 30322 USA; 2 Department of Oncology, The Affiliated Jiangyin Hospital of Southeast University Medical College, Wuxi, 214400 Jiangsu People’s Republic of China; 3 Tianjin Key Laboratory of Molecular Design and Drug Discovery, Tianjin Institute of Pharmaceutical Research, Tianjin, 300193 China; 4 Department of Neurosurgery, Winship Cancer Institute, Emory University, Atlanta, GA 30322 USA; 5 School of Public Health, Division of Health Systems, Management, and Policy, The University of Memphis, Memphis, TN 38152 USA

**Keywords:** AMD3100, Plerixafor, Leukemia, Breast cancer, Tumor

## Abstract

AMD3100, also known as plerixafor, was originally developed as an anti-human immunodeficiency virus (HIV) drug, and later characterized as a C-X-C chemokine receptor type 4 (CXCR4) antagonist. Previous reviews have focused on the application of AMD3100 in the treatment of HIV, but a comprehensive evaluation of AMD3100 in the treatment of leukemia, solid tumor, and diagnosis is lacking. In this review, we broadly describe AMD3100, including the background, functional mechanism and clinical applications. Until the late 1990s, CXCR4 was known as a crucial factor for hematopoietic stem and progenitor cell (HSPC) retention in bone marrow. Subsequently, the action and synergy of plerixafor with Granulocyte-colony stimulating factor (G-CSF) led to the clinical approval of plerixafor as the first compound for mobilization of HSPCs. The amount of HSPC mobilization and the rapid kinetics promoted additional clinical uses. Recently, CXCR4/CXCL12 (C-X-C motif chemokine 12) axis was found to be involved in a variety of roles in tumors, including leukemic stem cell (LSC) homing and signaling transduction. Thus, CXCR4 targeting has been a treatment strategy against leukemia and solid tumors. Understanding this mechanism will help shed light on therapeutic potential for HIV infection, inflammatory diseases, stem-cell mobilization, leukemia, and solid tumors. Clarifying the CXCR4/CXCL12 axis and role of AMD3100 will help remove malignant cells from the bone marrow niche, rendering them more accessible to targeted therapeutic agents.

## Background

In 1981, the US Centers for Disease Control and Prevention (CDC) published the first official report on the acquired immune deficiency syndrome (AIDS) epidemic. The identification of human immunodeficiency virus (HIV) as the causative agent for AIDS in 1985 spurred a massive search for inhibitors of the retrovirus. Rozenbaum et al. [1] reported in vivo efficacy of a polyoxometalate, HPA-23, that inhibited HIV levels in patients with AIDS. This evidence triggered a search for other analogues more effective in suppressing HIV levels. In 1990, De Clercq et al. [2] (the pioneer of AMD3100, from Rega Institute of Leuven, Belgium), in collaboration with investigators at Johnson Matthey, discovered bicyclam JM1657 (JM; Johnson Matthey) as an active anti-HIV agent. Attempts to re-synthesize JM1657 failed, but a new analogue of JM3100, later renamed AMD3100 (AMD; AnorMED, spun off from Johnson Matthey) was successfully synthesized in 1994 [3]. They found AMD 3100 inhibited HIV within a 1–10 nM range. The mechanism of action was thought to be the inhibition of viral replication at the time.

**Fig. 1:**
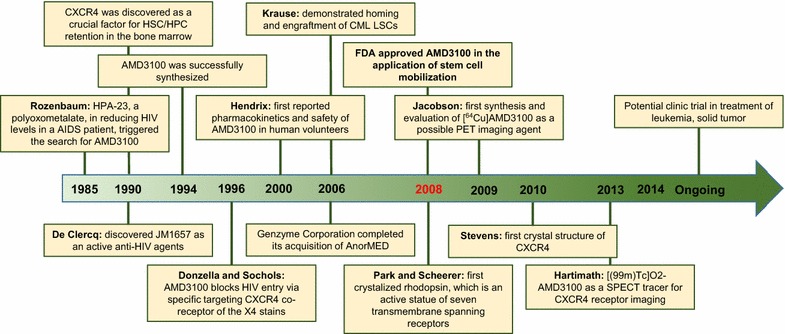
Timeline of major discoveries that shaped our understanding of AMD3100 and its practical use

Leading up to 1996, Donzella et al. [4] and Schols et al. [5] demonstrated that AMD3100 blocks HIV-1 entry and membrane fusion via specific targeting of the C-X-C chemokine receptor type 4 (CXCR4) co-receptor of the T-lymphotropic X4 strain (Fig. 1). It was not active against the M-macrophage tropic R5 strains, which are a CCR5-using population. These in vivo studies highlighted the effectiveness of AMD3100 against X4 HIV-1 infection. In 1996, Datema et al. [6] first demonstrated AMD3100 efficacy in severe combined immunodeficiency (SCID)-hu Thy/Liv mice infected with a clinical isolate of X4 HIV-1. Small animal in vivo results encouraged Phase I/II clinical studies. In 2000, Hendrix et al. [7] first reported the pharmacokinetics and safety of AMD3100 in human volunteers. These clinical data clearly showed AMD3100 effective in suppressing X4 HIV-1 levels in HIV-1-infected individuals.

During clinical trials, reduced X4 HIV-1 levels in HIV-infected volunteers were observed, as expected [8]. However, researchers noticed elevated white blood cell (WBC) counts. This unexpected observation was later translated in AMD3100 as an application to mobilize hematopoietic stem cells (HSCs). In 2006, Genzyme Corporation completed its acquisition of AnorMED (Fig. 1). Phase I studies demonstrated that AMD3100 had minimal side effects and effectively mobilized CD34^+^ HSC in both healthy volunteers and patients heavily pretreated with anti-tumor drugs. Phase II results built a compelling case of efficacy and safety. Two phase III, multicenter, double-blind, randomized, and placebo-controlled studies compared the safety and efficacy of AMD3100. Together, these trials provided sufficient evidence for the US Food and Drug Administration (FDA) approval of plerixafor (Mozobil, Genzyme Corp.; AMD3100) in 2008 (Fig. 1). AMD3100 proved an extremely specific and effective CXCR4 antagonist. However, AMD3100 is not limited to mobilization of HSCs. It is also used for a variety of disorders that depend on the interplay of CXCR4 with its agonist CXCL12 (also called: SDF-1) including leukemia and breast cancer.

## Molecular pharmacology of AMD3100 in blocking CXCR4

AMD3100 is strongly basic at physiological PH due to four primary amines in each cyclam ring. X-ray data show the protonated cyclam ring has a tendency to form complexes with carboxylic acid groups via hydrogen bonds [9].

**Fig. 2:**
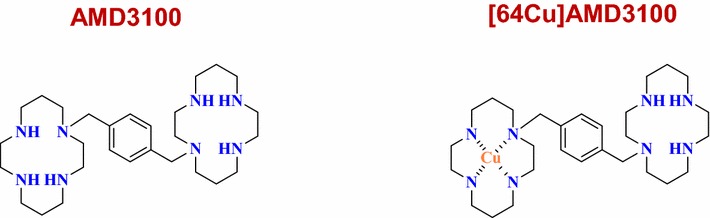
Chemical structure of AMD3100 and [64Cu]AMD3100

**Fig. 3:**
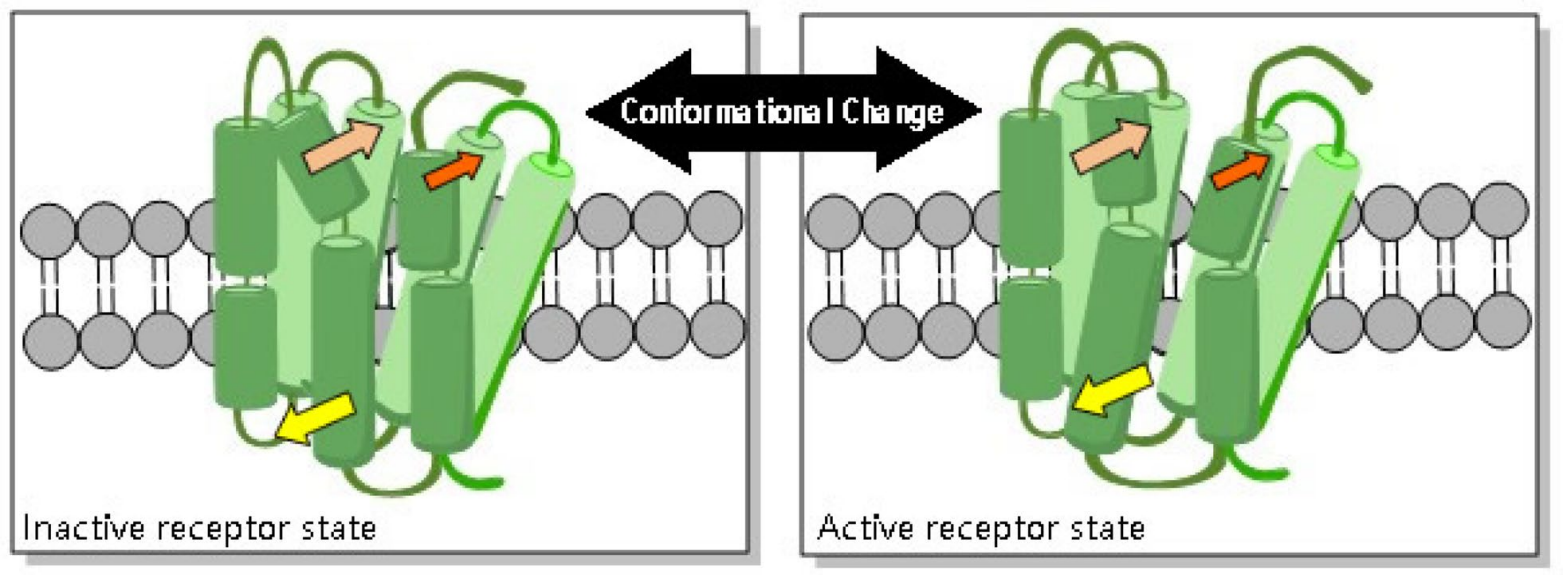
Schematic drawing of the 7TM receptor activation mechanism as proposed by the Global Toggle Switch Model

CXCR4 belongs to the largest family of proteins in the human genome, named seven transmembrane spanning receptors (7TM receptors) (Fig. 2a). Schwartz et al. [10] described activation of 7TM receptors in the Global Toggle Switch Model (Fig. 3). The crystal structure of the inactive and active states of CXCR4 was vital information in understanding 7TM receptor activation. In 2008, Park and Scheerer et al. [11, 12] provided strong evidence to the switch theory by crystalizing rhodopsin, presumed to be an active state representation. This crystal structure demonstrated that compared to the inactive state, the cytoplasmic half of transmembrane domain VI (TM-VI) is tilted outwardly away from the helical bundle by 6–7 Å. In 2010, the first crystal structure of CXCR4 was described by Stevens and coworkers [13]. The authors described the distinct differences between the structure of CXCR4 and other published crystal structure of 7TM.

Crystal structure data proved to be powerful, but limited in that they are snapshots of the inactive or active states, not sufficient when describing the dynamic process of activation. Therefore, functional receptor studies were needed. Several studies focused on the negatively charged domains of AMD3100 facing the binding pocket of CXCR4. In 2011, Gerlach et al. [14] performed a mutagenesis study of all aspartate residues facing the binding pocket of CXCR4. By testing all the mutants in competition binding, two aspartate residues were found in the extracellular ends of TM-IV (Asp^171^) and TM-VI (Asp^262^) associated with binding of AMD3100. These findings led to a proposal of the interaction model, in which AMD3100 spans the ligand-binding pocket and each cyclam ring binds to aspartate residues located at the extracellular ends of TM. Rosenkilde et al. [15] conducted comprehensive mutational substitutions of 16 residues located in TM-III, TM-IV, TM-VI, and TM-VII of CXCR4. They found AMD3100 not only depends strongly on two aspartate residues, as previously mentioned (Asp^171^ and Asp^262^), but also a glutamate (Glu^288^) in TM-VII. The combination of three acidic residues is a unique structure to CXCR4, which explains AMD3100′s high selectivity [16]. Given that mutational analysis did not reveal which side chains, or surrounding amino acids in proximity with the aromatic linker, are involved in AMD3100-interactions, a binding model of AMD3100 was suggested. The hypothesis suggests one cyclam ring interacts with the aspartate in TM-IV, while the other is located between the aspartate in TM-VI and the glutamate in TM-VII. The linker then acts as a constraint, preventing TM-VI from moving inward and into an active conformation.

## Clinical applications

### HIV entry inhibitor

To date, only two compounds have been approved by the FDA to modulate chemokines and their receptors. CCR5 antagonist maraviroc (Celsentri) is an HIV entry inhibitor approved in 2007. CXCR4 antagonist AMD3100 (Mozobil) was approved in 2008. Originally, AMD3100 was developed for treating HIV infections [17]. HIV enters the host cell through an interaction between envelope protein (gp120) and the host chemokine receptors, CCR5 and/or CXCR4. HIV that use CCR5 receptors are known as R5-tropic viruses, those using CXCR4 receptors are known as R4-tropic viruses, and those using both receptors are known as dual-tropic viruses. AMD3100 relies on the bicyclams to block the interaction between gp120 and CXCR4 [6]. The original clinical studies evaluated the efficacy of AMD3100 as an anti-HIV treatment modality [7]. However, continuous daily dosing requirements were impractical. Moreover, side effects were found including cardiac arrhythmia in two patients. AMD3100 is not currently being pursued for the treatment of HIV infections given dosing difficulty, side effects and lack of effect [18].

### Stem cell mobilization

Hematopoietic stem cell transplantation (HSCT) is increasingly used in the treatment of a variety of hematological malignancies and nonmalignant diseases such as sickle cell disease, autoimmune disease, and bone marrow failure. Currently, the use of mobilized peripheral blood HSC has replaced collection from pelvic bone marrow due to several reasons. First, the collection of peripheral HSC is less invasive. Second, there is a higher yield of HSC and progenitor cells collected from mobilized peripheral blood compared with bone marrow harvests. Third, there is significantly less toxicity and incidence of serious complications after peripheral blood stem cell transplantation (PBSCT) compared with bone marrow transplantation [19].

The mobilization procedure in normal donors requires administration of granulocyte-colony-stimulating factor (G-CSF). However, it causes significant side effects including bone pain, headaches and splenic rupture [20]. More importantly, G-CSF does not mobilize adequate numbers to safely undergo transplantation in a proportion of cases, particularly those with previous pretreatment of chemotherapy. AMD3100 is the only CXCR4 antagonist currently approved for HSC mobilization. Clinical data show less toxicity compared with HIV infections given the short time frame of administration of AMD3100 to initiate mobilization of hematopoietic stem and progenitors (HSPCs). Broxmeyer et al. [21] conducted the first proof-of principle studies on mobilization of HSPC in mice with AMD3100. The results were successfully translated by Dr. Dale’s group into human volunteers [22]. Mobilization of HSC and HPC in mice and humans is much quicker with AMD3100 (in minutes to hours) compared with G-CSF (days). Moreover, AMD3100 enhances mobilization of HPC in mouse models of genetic disease associated with “poor” response to G-CSF-induced mobilization [23].

The mechanism of AMD3100-induced mobilization involves the CXCR4/CXCL12 axis. The well-characterized mechanism of cell adhesion involves a CXCR4/CXCL12 interaction. This pathway was first discovered in the 1990s with a description of the chemokine CXCL12, and further explored in the hematopoiesis system [24, 25]. The CXCR4 is a G-protein-coupled receptor. After binding to its exclusive ligand CXCL12, CXCR4 activates several signaling cascades that trigger intracellular calcium release and phosphorylation of ERK 1/2. Current evidence indicates adhesion or mobilization of HSPC is dependent on CXCL12 concentrations in the blood and the bone marrow that act via CXCR4 signaling [26]. In addition, CXCR4 is considered more of a signaling receptor than an adhesion molecule. CXCL12 can be viewed as a chemokine directing cell movement. Dar et al. [26] also described CXCL12 homoeostasis in AMD3100-induced mobilization, showing AMD3100 administration reduced levels of CXCL12 signaling and the retention signals within the BM, also leading to an increased CXCL12-concentration in the peripheral blood. AMD3100 induced CXCL12 release is supported by in vitro results showing release of CXCL12 from osteoblasts and endothelial cells after incubation with AMD3100.

### Application of AMD3100 in leukemia

Hematopoietic cancer stem cells express the CXCR4 receptor. In conjunction with its CXCL12 ligand, CXCR4 mediates leukemia cell trafficking and homing to the bone marrow microenvironment in close proximity to marrow stromal cells. This provides a niche for leukemic cell growth and contributes to drug resistance. The use of CXCR4 antagonists may provide a novel therapeutic approach to interrupt these protective interactions and sensitize leukemia cells to chemotherapy in vivo.

#### Treatment of acute lymphoblastic leukemia

**Fig. 4:**
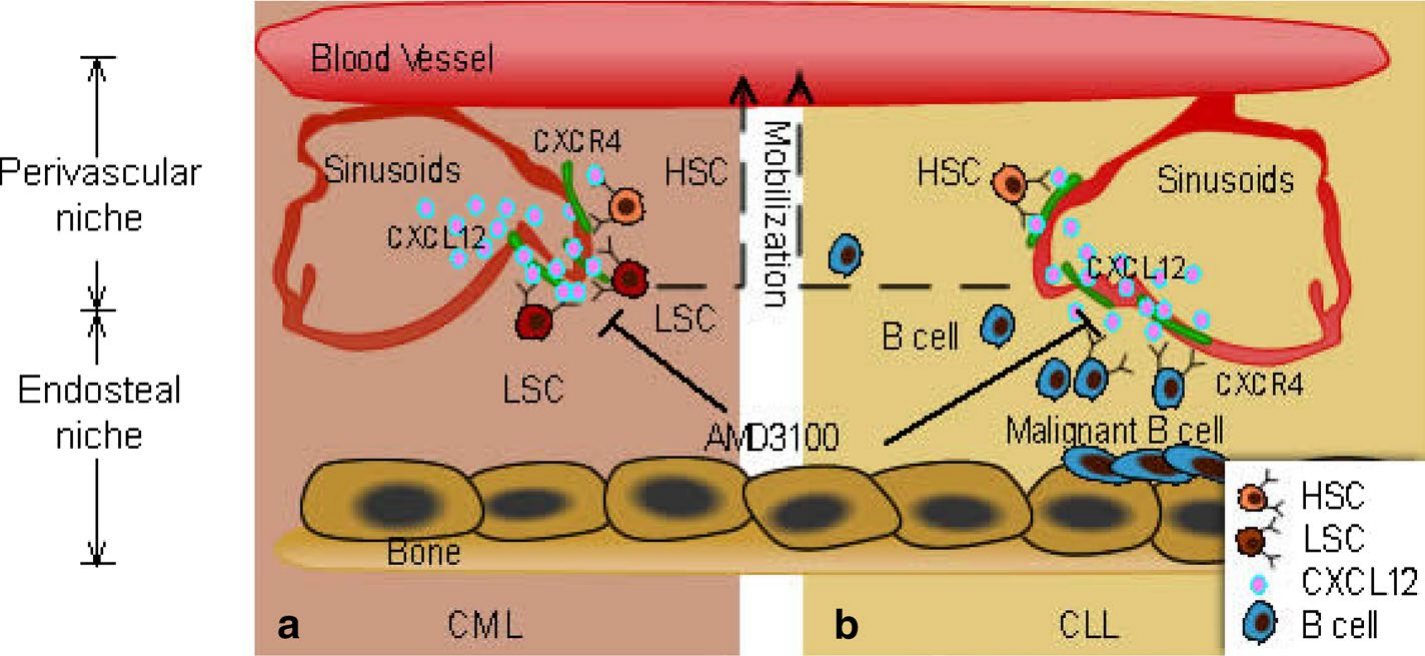
CXCR4/CXCL12 signaling in homing of hematopoietic stem cell (HSCs), CML leukemic stem cell (LSCs), and malignant B cells (i.e., CLL cells) to the bone marrow microenvironment. The perivascular niche is composed of a network of sinusoids (*red*) and adjacent reticular stromal cells niche (*green*) that constitutively secrete CXCL12. **a** In CML, LSCs (*red cells*) may exploit the same mechanism as HSCs (*brown cells*) to access niche. CXCR4/CXCL12-mediated interactions sheltered LSCs from the cytotoxic effects of TKIs. Interruption of CXCR4/CXCL12 interaction by AMD3100 may promote expulsion of quiescent LSCs from their niche and render them more accessible to targeted therapeutic agents. **b** In CLL, both HSCs and CLL cells express CXCL4, which directs chemotaxis of hematopoietic progenitor cells (HPC) and CLL from the circulation to the bone marrow. Malignant B cells (*blue cells*) utilize the CXCR4/CXCL12 axis to access niche that normally are restricted to HSC. This niche microenvironment will create favorable condition for their survival (such as fibronectin and hyaluronan) and provide survival, drug resistance signals to the CLL cells. Interruption of CXCR4/CXCL12 interaction by AMD3100 may promote expulsion of malignant B cells from bone marrow niche and render them more accessible to targeted therapeutic agents

Acute lymphoblastic leukemia (ALL) is characterized by monoclonal and/or oligoclonal proliferation of hematopoietic lymphoid precursor cells within bone marrow [27, 28]. Advances in childhood treatment have been achieved with over 80 % of individuals cured. However, a poor prognosis is expected for patients with different risk factors, such as relapses and central nervous system side effects [29]. Shen et al. [30] and Spiegel et al. [31] demonstrated down regulation of CXCR4 results in significant inhibition of ALL cell homing to the bone marrow (Fig. 4). CXCR4/CXCL12 axis plays an important role in the infiltration of extramedullary sites, which commonly express high levels of CXCL12, supported by the evidence of high expression of CXCR4 in all cells and extramedullary organ invasiveness [32]. In addition to the crucial role in migration, studies indicate that CXCL12 is involved in the pathogenesis of ALL, including facilitating metastasis, mobilizing tumor cells, and attracting of cancer stem cells within the tumor microenvironment [33, 34]. Moreover, CXCR4/CXCL12 signaling has been investigated extensively in activating multiple molecules, including PI3 K-Akt, MAPK and JAK-STAT signaling pathways [35]. Thus, it is reasonable that ALL subsets would benefit from the therapeutic strategy of CXCR4/CXCL12 axis. Indeed, Hatse et al. [16] showed that AMD3100 inhibited CXCR4 internalization and chemotaxis of ALL cells. Kato et al. [36] developed a therapeutic model where AMD3100 prevented relapse of extramedullary ALL cells after chemotherapy. These studies indicate that AMD3100 perturbs the maintenance of leukemic cells and/or leukemic stem cells in the bone marrow, prolonging exposure of chemotherapeutic agents in the peripheral blood. In addition, AMD3100 increases the proportion of leukemic cells in the circulation that are actively cycling [37].

#### Treatment of chronic lymphocytic leukemia

CXCR4 is expressed at high levels on the surface of peripheral blood chronic lymphocytic leukemia (CLL) cells [38]. Functionally, CXCR4 mediates CLL cell chemotaxis, migration across vascular endothelium, and migration beneath and underneath bone marrow stromal cells (BMSCs) that secrete CXCL12, termed “pseudoemperipolesis” [39, 40]. In addition, CXCR4 is involved in CLL cell migration and homing to tissue niches. Clinically, this function may be involved in drug-resistance and minimal residual disease (MRD), evidenced by the fact that CLL cells become highly resistant to conventional cytotoxic drugs when cultured in the presence of BMSC [41, 42]. Stromal cell niches in the bone marrow may provide protection where CLL cells are immune from chemotherapy, accounting for MRD and relapses after current CLL therapies [43]. Burger et al. [41] reported that CXCR4 antagonists inhibited CLL cell activation by CXCL12, and prohibited BMSC-mediated drug resistance. Therefore, the phase I trial of AMD3100 in combination with rituximab in CLL volunteers was initiated to determine the maximum tolerated dose (MTD) and the principal toxicities. Similar to AML, CML, and CLL, blockage of the CXCR4/CXCL12 pathway by AMD3100 is also being investigated in multiple myeloma and other hematopoietic cancers.

#### Treatment of acute myelogenous leukemia

The association of acute myelogenous leukemia (AML) cells with the bone marrow microenvironment may be mediated by CXCR4, which is invariably expressed on AML blasts [24]. Tavor et al. [44] found that pretreating human AML cells with neutralizing CXCR4 antibodies inhibited their homing and retention in the bone marrow of transplanted NOD/SCID/β2m mice. This suggests the CXCR4/CXCL12 axis is a target for anti-AML therapies. In a 122 AML patient chemotherapy study, Konoplev et al. [45] found high CXCR4 expression correlated with shorter overall survival and shorter event-free survival. Prompted by these observations, Uy et al. [46] conducted a phase I/II study of 52 patients with relapsed or refractory AML and determined the extent to which AMD3100 mobilized leukemic cells. These encouraging data promoted randomized phase III clinical trials in patients with relapsed AML to determine the role of CXCR4 blocking in improving complete remission rates in these high-risk patients.

#### The approach of combinational use of AMD3100 with MER kinase inhibitor to improve anti-tumor drug efficacy in the context of acute myelogenous leukemia

**Fig. 5:**
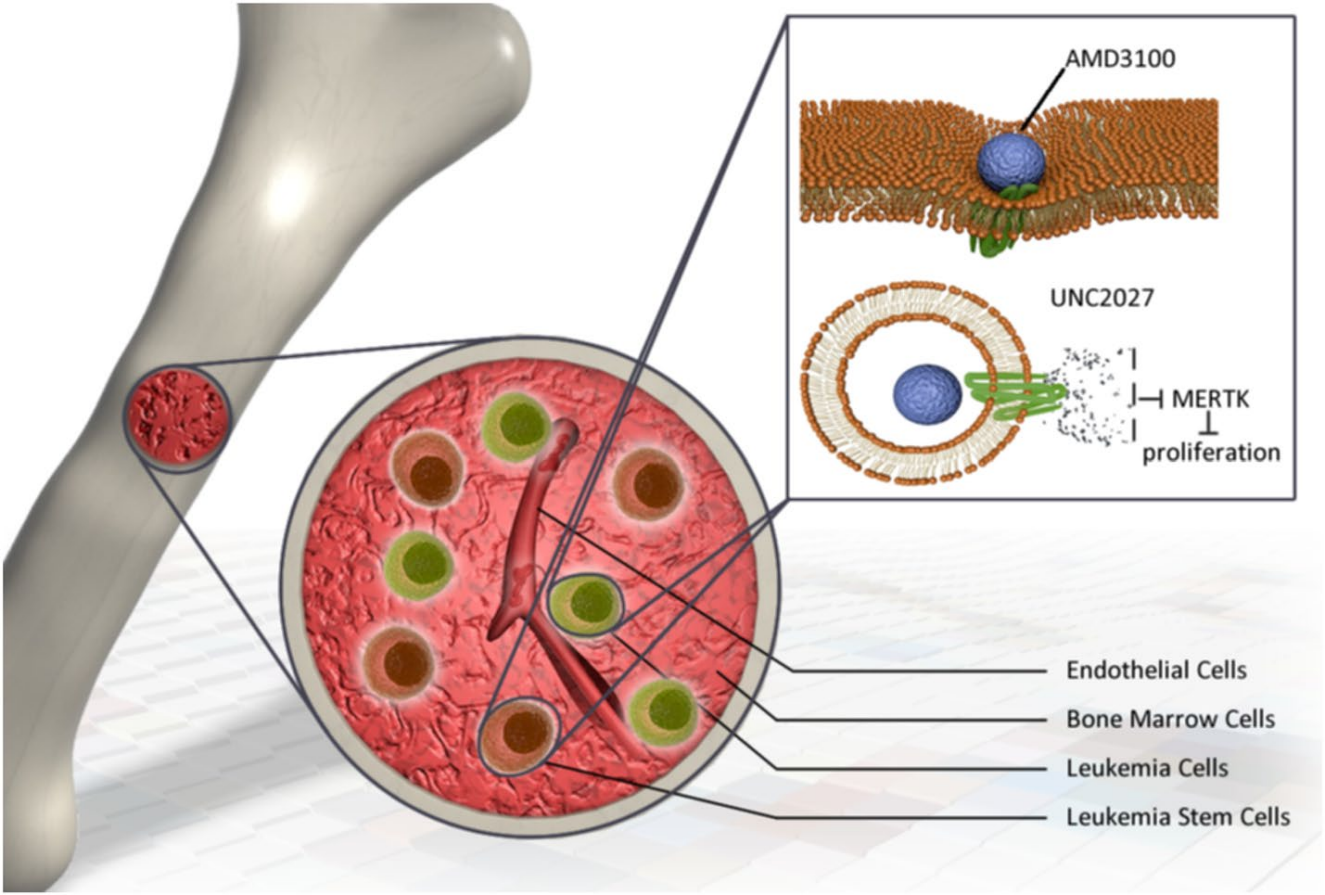
AMD3100 systemically deliver UNC2025 into bone marrow LSC and sensitize LSC to UNC2025 treatment

MERTK (TAM) family of receptor tyrosine kinases (RTKs) are aberrantly expressed in multiple haematological and epithelial malignancies. Targeting MERTK is a promising strategy in the treatment of AML. For example, the small molecule UNC2025, discovered by Dr. Graham, specifically targets MERTK and FMS-like tyrosine kinase 3 (FLT3) and was developed in preclinical phase for the treatment of ALL and AML [47]. However, chronic and sustained inhibition of TAM family RTKs have deleterious side effects, such as autoimmunity and retinitis. Relapse and drug resistance are the main obstacles for UNC2025. In order to reduce AML relapse, we expect AMD3100 to be combined with UNC2025 to optimize the drug efficacy. The following procedure has potential: couple UNC2025 with CXCR4-targeted lipid-coated ploy (lactic-co-glycolic acid (PLGA) nanoparticles (NPs) modified with a CXCR4 antagonist; AMD3100 will systemically deliver UNC2025 into bone marrow LSC and sensitize LSC to UNC2025 treatment (Fig. 5). AMD3100 attached to the NPs will also block CXCR4/CXCL12, reducing infiltration of tumor-associated macrophages, enhancing the anti-angiogenic effect.

#### Treatment of chronic myeloid leukemia

Tyrosine kinase inhibitor (TKI) based therapy has improved clinical outcomes in chronic myeloid leukemia (CML). Imatinib (Gleevec), an ABL TKI, is highly effective in newly diagnosed CML patients, with complete cytogenetic responses in more than 80 % of cases [48]. Although the targeted therapy elicits durable remissions it does not eradicate leukemic stem cells (LSCs), the reservoir of CML. In 2006, Krause et al. [49] demonstrated homing and engraftment of CML LSCs is characterized by dependence on the CD44 receptor and reduced reliance on CXCR4 and its ligand, CXCL12 (Fig. 4).

Given the reduced importance of CXCR4/CXCL12 in CML compared with normal HSCs, and CXCR4 antagonists are potential therapeutics for treatment of CML, the particular application that captures our attention is the recurrence of active leukemia. TKI-based therapy restores CXCR4/CXCL12 dependent trafficking and homing of CML LSCs to bone marrow [50]. Perturbation of the CXCR4/CXCL12 interaction with AMD3100 promotes egress of CML LSCs and progenitor cells from the bone marrow niche, potentially increasing their susceptibility to targeted strategies currently being evaluated in clinical trials.

#### Treatment of other type of leukemia

KMT2A gene, also known as mixed lineage leukemia (MLL) gene, encodes a lysine (K) specific histone methyltransferase 2A [51]. This enzyme contributes to the S-phase DNA damage checkpoint [52]. Rearrangements of the *MLL* gene were found in 5.2 % of all the AML cases and in 22 % of all cases [53]. Patients with MLL rearrangements have poor prognosis and shorter overall survival rates [54]. However, the mechanism leading to its rearrangement is still unclear. Given that half of patients recur with a short latency to relapse suggests that chemotherapy-resistant leukemia stem cells survive and recapitulate leukemia. The use of AMD3100 led to markedly enhanced efficacy with lestaurtinib [55]. Therefore, the bone marrow microenvironment is a mediator of chemotherapy resistance in MLL and targeting leukemia stem cell-niche cell interactions with AMD3100 would benefit the high-risk subtype of pediatric ALL.

Collectively, CXCR4 antagonists offer a new tool to mobilize leukemia cells from their protective bone marrow niche. AMD3100 is being explored in proof-of-principle studies in leukemia patients where leukemia cell mobilization can be assessed. Given the expression of functional CXCR4 receptors by a variety of other hematopoietic cancers and solid tumors, broader use of AMD3100 is the long-term goal.

### Application of AMD3100 in solid tumors

Breast cancer is the most commonly diagnosed malignancy in women worldwide [56]. Development of metastatic breast cancer is responsible for the majority of cancer-related deaths. Muller et al. [57] demonstrated that CXCR4 is expressed in human breast cancer cells and metastatic lesions. This evidence first identified a key function of CXCR4/CXCL12 in metastatic breast cancer. Many organs such as bone, lung, and liver express high levels of CXCL12, and are commonly affected by metastatic breast cancer. Kang et al. [58] injected human breast cancer cells in mice and found they preferentially formed bone metastases.

The CXCR4 signaling pathway facilitates breast cancer cell survival, proliferation, chemotaxis, invasion and adhesion. CXCL12 binding to CXCR4 stimulates the phosphatidylinositol-3-kinase pathway that subsequently activates the Akt pathway, facilitating growth factor-mediated cell survival and apoptosis suppression [59]. Akt also affects CXCR4 during proliferation of cells and migration toward chemotactic cytokine CXCL12 [60, 61]. In addition, mitogen-activated protein kinase pathway (MAP kinase pathway) is another signaling pathway regulated by CXCR4 that promotes proliferation and survival of cancer cells [62]. Moreover, CXCR4 in breast cancer activates Janus kinase/signal transducer and activator of transcription (JAK/STAT) pathway [63], src family [64], and angiogenesis [65]. Together, these pathways facilitate migration, invasion and metastasis.

These results are promising for developing a role for CXCR4 blockade in the treatment of breast cancer. A phase I/II study of AMD3100 in patients with breast cancer indicated preliminary signs of efficacy [66]. The use of AMD3100 in the treatment of bone metastases could be exploited in patients for targeted treatment with CXCR4 blockade. However, concern was raised regarding side effect on the normal hematopoietic balance in the bone marrow [67].

Studies also show that CXCL12 promotes pancreatic β-cell survival via activation of Akt [68]. These findings suggest AMD3100 agonists may prove beneficial for treatment of diabetes and lung cancer.

### Use of [64Cu]AMD3100 in molecular imaging of CXCR4 expressed tumors

CXCR4 is overexpressed in 23 different cancers [69]. Jacobson et al. [70] first showed the radiochemical synthesis and evaluation of [^64^Cu]AMD3100 as a possible PET imaging agent (Fig. 2b). They verified that [^64^Cu]AMD3100 was as potent AMD3100 as a CXCR4 inhibitor. Recently, [(67)Ga]-AMD3100 [71] and [(99m)Tc]O2-AMD3100 [72] were also developed as tracers for CXCR4 receptor imaging. However, the success of [^64^Cu]AMD3100-based imaging agents requires detailed characterization of the changes of CXCR4 and CXCL12 expression profiles in tumors, metastases, and normal tissue distributions.

## Conclusion

The development of bicyclam AMD3100 follows a meandering pathway, starting from a serendipitous discovery of an impurity in a commercial cyclam preparation to its original development as an HIV entry inhibitor, and finally leading to an FDA approved product, AMD3100, in the application of stem cell mobilization. Potential clinical applications of AMD3100 are currently being evaluated in several trials with great promise. The advantages of AMD3100 over other CXCR4 antagonists are listed in Table 1.

**Table 1:** Comparison of AMD3100 with other CXCR4 antagonists on the development

CXCR4 antagonist	Source	Target profile		Advantage	Citation
		Primary targets	Activity		
AMD3100	Compound	TM-IV (Asp^171^), TM-VI (Asp^262^), and TM-VII (Glu^288^)	IC50 = 2–20 nM	The only approved CXCR4 antagonist; no species specific, thus studies of AMD3100 in mouse, canine, monkey, and cell lines have translated into human clinical studies quickly	De Clercq [2, 3, 17, 18]; Hatse et al. [16]
EPI-X4	Endogenous peptide	The positively charged face of the ring of EPI-X4 interacts with the negatively charged extracellular face of CXCR4	IC50 = 8.6 ± 3.1 µM	Endogenous antagonist; no cytotoxity	Zirafi et al. [74]
KRH-3955	Compound	Binding sites of KRH-3955 are located in a region composed of all three extracellular loop (ECLs) of CXCR4	IC50 = 0.61 nM	Administered orally with much more potent anti-HIV-1 activity than AMD3100 and KRH-1636	Murakami et al. [75]
POL5551	Compound	Similar to plerixafor, POL5551 bound to the extracellular loops but not to the N-terminal moiety recognized by 1D9	IC50 of 12G5 binding at 1 h ≤2.5 nM	POL5551 is a potent antagonist of surface CXCR4 in pre-B and T cell ALL cell lines	Karpova et al. [76]; Sison et al. [77]
LY2510924	Compound	LY2510924 occupied a binding pocket and possessed ligand–receptor interactions with CXCR4 residues such as Asp187, Arg188, Gln200, His113, and Tyr190	IC50 = 0.079 nM	Phase II clinical studies for cancer	Peng et al. [78]

The success of AMD3100 follows the typical pathway of drug discovery process that involves identification of target, screening process, lead development, and then preclinical trials. The following steps are essential to fulfill the final approval of AMD3100. First, an illustration of a detailed molecular pharmacology of AMD3100 in blocking CXCR4 is needed. Second, an understanding of the role of CXCR4/CXCL12 axis interaction in the HSC’s homing and engraftment is demonstrated. Thus, AMD3100 provided a novel strategy to enhance donor cell engraftment and recovery following transplantation. Third, understand how the CXCR4/CXCL12 axis is involved in tumor functions, such as how LSC homing and signaling transduction helped translate findings into additional clinical uses in leukemia and solid tumors.

AMD3100 has demonstrated clinical efficacy in the context of mobilization of HSCs and HPCs, and triggered other clinical uses with great promise. One critical reason is that AMD3100 is not species specific, thus studies of AMD3100 in mouse, canine, monkey, and cell lines have translated into human clinical studies quickly. However, researchers need to consider that circadian rhythms influence circulating levels of HSCs and HPSs [73]. It is critical to control the timing of AMD3100 administration in the context of therapeutic use. In addition, side effects after long-term administration add potential problems. Shorter-term usage and lower dose would be fundamental keys to the success in clinical use.

## Abbreviations

AIDS: acquired immune deficiency syndrome
AML: acute myelogenous leukemia
BMSC: bone marrow stromal cell
CDC: Centers for Disease Control and Prevention
CLL: chronic lymphocytic leukemia
CML: chronic myeloid leukemia
CXCR4: C-X-C chemokine receptor type 4
FDA: Food and Drug Administration
G-CSF: granulocyte-colony-stimulating factor
HIV: human immunodeficiency virus
HPC: hematopoietic progenitor cell
HSC: hematopoietic stem cell
HSCT: hematopoietic stem cell transplantation
JAK: Janus kinase
LSC: leukemic stem cell
MRD: minimal residual disease
MTD: maximum tolerated dose
PBSCT: peripheral blood stem cell transplantation
SCID: severe combined immunodeficiency
STAT: signal transducer and activator of transcription
TKI: tyrosine kinase inhibitor
